# Removal of the Harmful Nitrate Anions from Potable Water Using Different Methods and Materials, including Zero-Valent Iron

**DOI:** 10.3390/molecules27082552

**Published:** 2022-04-14

**Authors:** Hany M. Abd El-Lateef, Mai M. Khalaf, Alaa El-dien Al-Fengary, Mahmoud Elrouby

**Affiliations:** 1 Department of Chemistry, College of Science, King Faisal University, P.O. Box 400, Al-Ahsa 31982, Saudi Arabia; mmkali@kfu.edu.sa; 2 Chemistry Department, Faculty of Science, Sohag University, Sohag 82425, Egypt; alaa.eldaly2@yahoo.com; 3 Faculty of Science, King Salman International University, Sinai 46612, Egypt

**Keywords:** potable water, treatment, nitrate removal, zero-valent iron, electrochemical method, physical adsorption method, redox reaction method

## Abstract

Drinking water containing nitrate ions at a higher concentration level of more than 10 mg/L, according to the World Health Organization (WHO), poses a considerable peril to humans. This danger lies in its reduction of nitrite ions. These ions cause methemoglobinemia during the oxidation of hemoglobin into methemoglobin. Many protocols can be applied to the remediation of nitrate ions from hydra solutions such as Zn metal and amino sulfonic acid. Furthermore, the electrochemical process is a potent protocol that is useful for this purpose. Designing varying parameters, such as the type of cathodic electrode (Sn, Al, Fe, Cu), the type of electrolyte, and its concentration, temperature, pH, and current density, can give the best conditions to eliminate the nitrate as a pollutant. Moreover, the use of accessible, functional, and inexpensive adsorbents such as granular ferric hydroxide, modified zeolite, rice chaff, chitosan, perlite, red mud, and activated carbon are considered a possible approach for nitrate removal. Additionally, biological denitrification is considered one of the most promising methodologies attributable to its outstanding performance. Among these powerful methods and materials exist zero-valent iron (ZVI), which is used effectively in the deletion process of nitrate ions. Non-precious synthesis pathways are utilized to reduce the Fe^2+^ or Fe^3+^ ions by borohydride to obtain ZVI. The structural and morphological characteristics of ZVI are elucidated using UV–Vis spectroscopy, zeta potential, XRD, FE-SEM, and TEM. The adsorptive properties are estimated through batch experiments, which are achieved to control the feasibility of ZVI as an adsorbent under the effects of Fe^0^ dose, concentration of NO_3_^−^ ions, and pH. The obtained literature findings recommend that ZVI is an appropriate applicant adsorbent for the remediation of nitrate ions.

## 1. Introduction

One of the most stable forms of nitrogen oxidation states’ compounds is nitrate (NO_3_^−^), which is difficult to reduce and is a naturally existing ion as a portion of the nitrogen cycle. Although it is chemically inert, it can be degraded by microbial processes [1]. Nitrates are present in groundwater due to the high nitrogen fertilizer use and the oxidation of ammonia-containing wastewater [2]. In soil, organic wastes and inorganic fertilizers containing nitrogen compounds are initially degraded to yield ammonia and turned into nitrate and nitrite [3,4].

The most hazardous effect of nitrite ions on humans is their role during the oxidation process of hemoglobin into methemoglobin, which cannot carry oxygen to the body’s skin. The diminutive transportation process of oxygen can be clinically detectable once methemoglobin concentration reaches 10% of the normal hemoglobin concentration in the human body. This so-called methemoglobinemia results in cyanosis and at greater doses causes asphyxia. The natural methemoglobin scale in adults is lower than 2% and in kids under three months below 3% [2]. In Canada and the United States, the maximum allowable nitrate content for drinkable water is 45 mg/L, although the World Health Organization (WHO) and the European Community have placed the limit at 50 mg/L [5].

Nitrate concentration in water can be determined by several techniques such as a spectrophotometric technique [6], high-performance liquid chromatography (HPLC) [7], ion chromatography [8], short-column ion-pair chromatographic separation [9], ion-interaction liquid chromatography [10], and electrochemical determination, including voltammetric detection [11,12].

Ion exchange, reverse osmosis, biological denitrification, and chemical denitrification are some of the approaches for removing nitrate from aqueous solutions. Chloride ions interact with nitrate ions from the resin in the ion-exchange process [13,14,15]. Reverse osmosis (RO) technology removes nitrates by forcing raw water through a semi-permeable membrane that allows water to flow through while retaining most of the dissolved minerals. However, this method has significant drawbacks, including high installation and maintenance costs. Moreover, it needs pretreatment, low water efficiency (10–25%) for low-pressure applications, membrane monitoring, and continuous maintenance [16]. The biological denitrification of water has been widely employed, and numerous species such as bacteria, fungus, protozoa, and microalgae have been used in this process [17]. For chemical denitrification, nitrates are chemically reduced using several chemical reducing agents such as active metals and alloys (Cd, Cd amalgam, Devarda’s alloy (50% Cu, 45% Al, 5% Zn), Zn, and Arndt’s alloy (60% Mg, 40% Cu)). Additionally, ammonia, borohydride, format, and other organic species were used. Hydrazine and hydroxylamine, hydrogen, and iron (II) were also utilized. In addition, many techniques have been used to reduce nitrates such as electrochemical, photochemical, and thermal [18]. Nano ZVI is considered a powerful and available reducing agent, which can swap harmful chemicals for harmless ones. It can also be used to accelerate the reduction of carcinogenic and toxic metals such as chromium (Cr) from an oxidation state (VI) to a more stable state (III) [19].

Because nitrate in water is one of the most toxic components, the main purpose of the present review is to describe many protocols that are applied in the remediation process of nitrate anions from aqueous solutions, showing different synthesis pathways followed by describing earlier studies and techniques used to enhance the properties for this purpose. After thoroughly investigating these methods, it was found that nitrate removal can be accomplished by selecting a low-cost, effective, renewable, and high-quality nitrate removal technology with no side products. The adsorption and reduction of nitrate on the adsorbent surface are some of these protocols, and ZVI nanoparticles are considered a successful candidate adsorbent for nitrate removal.

## 2. Electrochemical Methods of Nitrate Removal from Water

There are numerous routes for reducing NO_3_^−^ ions from aqueous solutions, and these methods vary in conditions and chemical reactions as follows.

### 2.1. Sulfamic Acid and Zinc Metal Method

In this method, nitrate samples are added to a chemical reactor consisting of a reaction vessel, agitator, and an electrolytic cell [20]. This reactor is in contact with two chemical reagents (sulfamic acid and Zn metal) [21]. Here, nitrate is reduced to nitrogen gas according to the following reaction [22,23]:

NO_3_^−^ + Zn + H^+^ + H_3_NSO_3_ → N_2_ + SO_4_^2−^ + Zn^2+^ + 2H_2_O (1)

Zn cations are subsequently reduced to zinc metal and re-involved in the reaction as a result of the usage of zinc metal as a catalyst in this process. On the other hand, sulfamic acid is the reagent that is consumed.

Once water containing nitrate is fed to the reactor, the reaction starts immediately and releases nitrogen gas bubbles. The pH is an important parameter in this reaction. After a few seconds, the water pH is immediately decreased from sulfamic acid addition for all conditions. Then, the treated water pH is increased to 6.3–7.3. The increase in pH value is due to the electrochemical reactions that consume the H^+^ ions in the electrolytic cell [20].

### 2.2. Electrochemical Denitrification Method

The temperature effect of the electrolyte, electrode type, pH, and current density are all important factors in electrochemical denitrification [24]. The electrode material is another factor that might influence the effectiveness of anodic oxidation of contaminants [25]. Thus, Al, Cu, Fe, Cu-Zn, and stainless steel have been applied as promising cathodes [26,27,28]. Stainless steel, Fe, and Cu are successfully used as cathodic electrodes in the electro-reduction process of NO_3_^−^ ions being converted into N_2_ gas [27,28,29,30]. Aluminum, iron, and titanium electrodes have a larger electron-donating capacity, explaining their better nitrate removal effectiveness [31].

The nitrate reduction efficiency was poor in cyclic voltammetric tests using graphite electrodes [32]. Gender et al. claimed to have achieved high nitrate reduction efficiencies (up to 75%) using graphite in a divided electrochemical cell containing strong basic nitrate and nitrite electrolytes. This due to the absence of anodic interference or nitrite anodic oxidation [33]. Many electrodes have used to reduce nitrate; some are explored in detail below [34].

#### 2.2.1. Fe Electrode

The rate of nitrate–nitrogen transformation utilizing Fe as a cathode is almost 80% at pH 7 and pH 9 after 5 h. Under these conditions, the generation of ammonia is extremely high, and nitrite concentrations are extremely low. It was noticed that at pH 9, the total nitrogen content drops dramatically, but at pH 7, it remains unchanged [31]. The presence of ammonium ions in water, which is a function of pH, might explain why total nitrogen concentrations drop at pH 9 [35]. In the previous approach, with the existence of 0.30 and 0.50 g/L of NaCl, the nitrate–nitrogen transformation dropped from 100.0 to 9.0 and 12.9 mg/L in 180 min, respectively. The rate of nitrate reduction was slower at high NaCl doses than at low Na dosages. The rate of nitrate reduction is slow in the presence of chloride ions [27]. Additionally, the ability of chloride ions to be adsorbed on the electrode surface explains its efficiency in poisoning the active centers of nitrate reduction; thus, they may reduce the functional centers of the electrode surface area. Consequently, the selectivity of the sorbed chloride ions limits the NO_3_^−^ reduction [36]. Another explanation for the low rate is that chloride ions and NO_3_^−^ ions compete for mobility towards the electrode because Cl^−^ ions are smaller than NO_3_^−^ ions, allowing Cl^−^ ions to penetrate the electrode pores and promote corrosion.

In a dual-chamber cell, electrochemical reduction of nitrate was investigated utilizing Fe as a cathode and a Ti/TiO_2_ nanotubes array as an anode. The efficacy of various cathodic electrodes for the elimination of NO_3_^−^ was in the sequence Fe > Al > Cu > Ni. When utilizing Fe as a cathode, the elimination of nitrate (99.8%) in a dual-chamber cell was substantially greater than that in a single-chamber cell (84.3%) in 2.5 h. The maximum (99.8%) nitrate removal was attained in a dual-chamber cell with a current density of 15 mA/cm^2^. The reduction process of NO_3_^−^ was deduced as a first-order reaction. The reduction rate constant dropped from 2.2 h^−1^ (100.0 mg/L) to 0.9 h^−1^ (1000.0 mg/L) when the starting concentration of nitrate was increased, although a significant NO_3_^−^ elimination of 88.30 percent was still obtained with 1000.0 mg/L NO_3_^−^ ions. Concurrently, NO_3_^−^ reduction and the oxidation of bi-products of NH_3_ and NO_2_^−^ were accomplished in a dual-chamber cell using an Fe cathode in the existence of 500 mg/L NaCl by NO_3_^−^ removal efficacy of 94.3 percent without byproducts as distinguished through the electrolyte [37].

#### 2.2.2. Aluminum Electrode

In comparison to iron electrodes, the majority of nitrate is converted to ammonia on aluminum electrodes, resulting in a larger proportion of nitrite generation [31]. T. Brahmaiah et al. employed an aluminum electrode and altered parameters such as electrode size, the distance between anode and cathode, and applied potential. The results of electrochemical-coagulation (EC) batch experiments showed that the performance of the EC reactor varied with different operating conditions. The optimum operating conditions were detected by performing a series of compact experiments using aluminum electrodes at different applied potentials of 5, 10, 12, 14, and 16 V. The optimum voltage was found to be 14 V, using two electrodes of 5 × 5 cm^2^ and 7 × 7 cm^2^ dimensions for an electrolysis time of 120 min, and the spacing between the electrodes was 2.5 cm and 0.5 cm. The efficiency of the nitrate–nitrogen removal was found to be 76% by using the electrode of 7 × 7 cm^2^ size at 14 V. It is noticed that at higher voltages, the removal had good efficiency, but the formation of sludge was increased [38].

#### 2.2.3. Copper Electrode

The voltammetric behavior was achieved within a potential from −0.2 V to −1.6 V, in which copper was used as the cathode. The addition of nitrate ions produces two current waves in a more negative potential area of hydrogen evolution overpotential [32]. On the other hand, the copper electrode submerged in 0.1 M HClO_4_ does not show any peaks in the linear sweep voltammetric behavior (LSV). However, in the presence of nitrate in 0.1 M HClO_4_, the LSV of the copper electrode exhibits a significant rise in cathodic current due to the reduction of nitrate. This suggests that there are two charge transfer stages in the electrochemical reaction. At −0.6 V, nitrate is converted to nitrite, which is subsequently converted to NO at −0.7 V [39].

In previous research, the electrochemical reduction of nitrate on a copper cathode in 0.1 M NaOH electrolyte was accomplished utilizing cyclic voltammetric (CV), chronoamperometric (CA), and coulometric techniques. The obtained data reveal that the electro-reduction of NO_3_^−^ ions on the copper electrode in the basic media is a complicated catalytic method featuring a nonreversible electron transfer followed by an adsorption process. The electrochemical reduction process of nitrate consists of three charge transfer phases, each corresponding to a distinct potential range for synthesizing NO_2_^−^, N_2_, and NH_4_^+^. The creation of N_2_ in the second potential region aids the electrochemical reduction technique to eliminate pollutants that exist in polluted aqueous solutions containing NO_3_^−^ [40].

In an acidic aqueous solution, polypyrrole was applied as a surface coating on the copper electrode (PPy–Cu), and nitrate concentrations ranging from 5 to 200 mM were used. The imposed voltages versus Ag/AgCl were −0.4, −0.6, −0.8, −1.0, and −1.2 V. It was discovered that changing the electrocatalyst produced the same results but at different concentrations. At practically all applied potential levels, the nitrite ion is produced on the bare Cu electrode. However, the modified PPy–Cu electrode is characterized by the production of ammonia only at potentials between –0.4 and –0.8 V. In the case of the PPy–Cu electrode as an electrocatalyst, around 33% of the nitrate was transformed exclusively into ammonia, with no nitrite detected [41]. In addition to the in situ and digital analysis techniques, the electrochemical methods were used to investigate the electrocatalytic reduction of NO_3_^−^ on copper single crystals. This shows how the NO_3_^−^ reduction on copper single crystals is affected by the pH value and surface construction of the electrolyte. A Cu (111) cathode converts NO_3_^−^ to NO_2_^−^ at potentials below the Cu (100) cathode in an alkaline medium. Cu (100), on the other hand, lowers nitrite to hydroxylamine even more. Although the CV profiles differ in acidic media, both copper electrodes produce identical intermediates and products: NO, NO_ads_, and ammonium ions. Furthermore, in both alkaline and acidic electrolytes, the passivation of Cu (111) during nitrate or nitrite reduction has a stronger impact than the passivation of Cu (100). The suppression of the electrocatalyst is characterized as presumptive because of the establishment of H_ads_. Therefore, the variance among the degree of de-activation of such two surfaces pertains to the greater H_2_ evolution efficiency of the Cu (111) electrode. Hence, NO_3_^−^ reduction using Cu electrode could occur under pH– control process, producing NO and ammonium ions in acidic solutions and NO_2_ and NH_2_OH in basic solutions [42].

#### 2.2.4. Tin Electrode

The electrochemical reduction of NO_3_^−^ ions using tin cathodes in the range of high cathodic voltages was examined in 0.1 M K_2_SO_4_ and 0.05 M KNO_3_ solutions. The great efficiency of NO_3_^−^ reduction with an excellent selectivity percentage (%S) of N_2_ (92.0%) was achieved at −2.9 V versus Ag/AgCl. The most prevalent byproducts were NH_3_ (8.0%) along with NO_2_^−^ (<0.02%). Small quantities of N_2_O and traces of NO were also obtained. When the cathodic voltage rises, N_2_ selectivity increases, whereas NH_3_ selectivity exhibits a maximum at −2.2 V. It is noticed that the selectivity (%S) for NO_2_^−^ diminishes from 65% at −1.8 V to <0.02% at −2.4 V. The kinetic analysis showed the generation of N_2_ and NH_3_ yields within the NO_2_^−^ intermediate. The reduction reaction obeys first-order kinetics for both NO_3_^−^ and NO_2_^−^ at cathodic potentials above −2.4 V. Initially, the Faradaic efficiency (%FE) of the reduction at −2.9 V was ~60%, which dropped to 22% after 40 min. Cathodic corrosion of Sn was detected, which was more intense in the deficiency of NO_3_^−^. Small quantities of Sn hydride were discovered in the voltages with more negative than −2.4 V [43].

The nitrate reduction was prolonged to entire cells by the same effect even as it was in the divided cells. The reduction is aided by alkali and the use of tiny quantities of tin (II) salts, which are involved in a dynamic exchange between cathodic deposition and cathodic corrosion. There may be a precedence in the literature for tin (II) salts to perform as catalysts to enhance the hyponitrite “dimerization” step rather than ammonia reduction. The number of hyponitrite ions (N_2_O_2_^2−^) decreases when using an electrolytic technique, which beats the interception of these particles as a reaction intermediate, resulting in predominantly N_2_O rather than N_2_ as a result of thermal degradation [44]. The effect of nitrate concentration within the range of 100–62,000 mg/L NaNO_3_ in NaCl solutions was investigated at a fixed voltage of −2.8 V versus Ag/AgCl. It was noted that the reduction rate follows Langmuir–Hinshelwood kinetics. At concentrations above 18,600 mg/L, the reaction obeys zero-order kinetics, whereas, at lower concentrations, the reaction obeys first-order kinetics. It was also discovered that as the nitrate concentration increases from 100 to 1500 mg/L the nitrogen selectivity rises from 70 to 83% and is still nearly constant at higher concentrations of NO_3_^−^ ions, whereas S% of NH_3_ is the opposite, diminishing from 25.0 to 11.0%. The% FE is raised with concentrations of NO_3_^−^ ions increasing from 25% at 6200 mg/L to 78%, and at 62,000 mg/L during the reduction to 95.0% of nitrate ions. At the higher concentration of NO_3_^−^ ions, hyponitrite and hydroxylamine were observed as reaction intermediates. The evolution of hydrogen, in the given reaction conditions, occurs during cation reduction of the supporting electrolyte rather than the Volmer-Tafel mechanism, and then the NO_3_^−^ reduction takes place during the electrochemical hydrogenation [45].

In addition, the effects of the voltage, the concentration, and the nature of the supporting electrolyte on the nitrate reduction efficiency with tin cathode were investigated by both potentiodynamic and potentiostatic techniques. The reduction rates of NO_3_^−^, producing N_2_, are increased when the voltage increases from −1.8 to 2.8 V versus Ag/AgCl, whereas by forming nitrite, the voltage is diminished. The formation of NH_3_ exhibits a maximum at −2.4 V, and then it reduces. The reduction rate of nitrate at −1.8 V versus Ag/AgCl increases as NaCl concentration rises. The supporting electrolyte cation increases the rate of reduction through the series Li^+^ <Na^+^ <K^+^ <Cs^+^ due to the presence of NH_4_^+^, and with multivalent cations such as Ca^2+^ and La^3+^, higher rates than those of the alkali metals have been reported. At a potential of −1.8 V, the supporting electrolyte anion slows down the reduction rate by this sequence: I > Br > Cl > F. The Frumkin theory, as well as the hypothesis of ion-pair creation between the supporting electrolyte cation and the reacted nitrate, can interpret these observations qualitatively [46].

## 3. Adsorption Method

The adsorption process is described as the process in which the soluble compounds in the solution are gathered on a suitable barrier [47]. It is well understood that the adsorbent surface area must be large for the activation process to enhance it [48]. In the existence of hardness induced by Ca(HCO_3_)_2_, nitrate removal is significantly reduced. The impact of these particles on nitrate removal can be explained by the fact that NO_3_^−^ and HCO_3_^−^ ions have similar structures. The angles between the C–O and N–O bonds are matching and equal to 120.0° in each of these ions. It can therefore be supposed that HCO_3_^−^ anions adsorbed to the same energetic centers on the rice straw RS(ox.) carbon-oxidized surface [49]. It is well-known that the activated carbon resulting from ecofriendly waste with a large content of carbon is the most significant compound for removing eco-friendly pollution (liquid and gas contaminations). Therefore, ecological wastes are very significant precursor compounds for synthesized ready-activated carbon. Numerous polymeric materials supporting crude, lignocellulosic (agriculture bi-products), and coals are traditionally utilized as a starting substance for synthesized activated carbon [50]. Sodium hydroxide is used for commercial granular-activated carbon modification accompanied by a cationic surfactant to promote its efficacy for nitrate removal from water. The blend of both reagents can enhance nitrate removal efficiency, which is generally attributed to the alteration by cationic surfactant; in contrast, the specific surface area, and porous volume are not important factors in nitrate removal. Additionally, the adsorption capacity of activated carbon towards nitrate was established to be independent of pH. The equilibrium time and optimum adsorbent amount were found to be 120 min and 4000 mg/L, respectively. The maximum adsorption ability of nitrate, based on the Langmuir model, was found to be 21.52 mg/g. It was also found that the adsorption was a pseudo-second-order kinetic type [51].

Composites of the Fe_2_O_3_ nanoparticles and activated carbon (Fe–AC) have high efficiency for nitrate removal, due to enhanced features towards the remediation process compared with activated charcoal. Based on response surface methodology data at optimal conditions, AC might accomplish 69% of nitrate elimination after a contact time of one hour, when the initial dose of the nitrate is 147.32 mg/L and at pH = 3. Additionally, Fe–AC can remove nitrate with an efficiency of about 96% after one, but at a pH of 5.1 and an initial dose of 69.16 mg/L. The data of adsorption equilibrium agreed well with the isotherm model of Langmuir, and the kinetics adsorption data followed the pseudo-second-order reaction [52]. The derived activated carbon from sugar beet pulp was found to possess high pore volumes. It was discovered that the net pore volume and the specific surface area of the fabricated activated carbon were 0.966 cm^3^/g and 1826 m^2^/g, respectively. When using this activated carbon to remove nitrate from wastewater, the pH value did not affect the nitrate removal. Therefore, the kinetics illustrate that the pseudo-second-order equation provides a greater correlation for the adsorption phenomena. The model of Langmuir was found to support the simplest work of the empirical findings. It was established that the adsorption ability was augmented by increasing the solution temperature [53].

Some studies found that activated carbon adsorbents made from rice husk and paper sludge had a great potential for adsorbing nitrate. In addition, using ZnCl_2_ as an additive significantly impacts nitrate adsorption levels. The rate of nitrate adsorption increases as the adsorbent concentration rises. The adsorption by such an adsorbent is associated with the pseudo-second-order kinetic model and the Langmuir adsorption isotherm in these investigations [54]. The activated carbon material is superior to Clinoptilolite and has more tendency to remove nitrate. The most effective potency is 63% for removing nitrate from 60 mL water for activated carbon in a 4 g dosage sorbent, a temperature of 293 K, contact time of 60 min, pH = 6.5, and the initial dose of 100 mg/L, and from the adsorbent Clinoptilolite, for a contact time of 60 min, pH = 5.5, 4 g adsorbent dosage, the temperature of 293 K, and the initial concentration of 100 mg/L, the most effective potency is 9%. Freundlich isotherm for activated carbon and the Langmuir adsorption model showed good agreement for the Clinoptilolite adsorbent. It was found that the first-order pseudo adsorbing kinetics are a good match for Clinoptilolite and a second-order pseudo adsorbing kinetics for the activated carbon [55].

Balasundaram et al. used chitosan as an adsorbent for nitrate removal; it is known that chitosan is a nitrogenous polysaccharide made from acetyl glucosamine and glucosamine units. In addition to the use of chitosan for nitrate removal, it was found that chitosan in the solution can remove hardness and chlorides as well as nitrate reduction [56]. Chitin is also used as a natural adsorbent for nitrate removal. The adsorption kinetics of nitrate ions on chitin are very fast compared with other adsorbents, and equilibrium is reached after 2 min contact time for an initial concentration of about 100 mg/L and after 10 min for an initial dose of about 1000 mg/L [57].

It has been reported that modified zeolite is a good adsorbent that is used for nitrate removal. Surfactant modified zeolites (SMZ) with diverse exposure types were prepared by adding the cetyl pyridinium bromide (CPB) onto the surface of the natural zeolite. SMZ and natural zeolite with CPB monolayer coverage were insufficient for nitrate removal from the aqueous medium. However, SMZ with an irregular bilayer or bilayer CPB coverage was effective in nitrate removal, and the adsorption capability of nitrate by SMZ was increased with its CPB support. For distinctive SMZ coverage with bilayer CPB, the adsorption process of nitrate followed the pseudo-second-order kinetic model. Freundlich, Langmuir, and Dubinin–Radushkevich (D–R) isotherm models agreed well with the obtained results [58]. The adsorption capacity for surfactant modification of zeolite using hexadecyltrimethylammonium bromide increased, and the empirical findings indicated that nitrate adsorption onto SMZ is exothermic. An upsurge in adsorbent quantity resulted in a consistent upsurge in the nitrate removal percentage from wastewater samples [59]. Activated perlite is considered one of the most effective sorbent materials for removing nitrate from aqueous solutions. Perlite gives high efficiency for removing nitrate at the optimum conditions: at a contact time of 120 min, pH 5, and an adsorbent quantity of 0.7 g, the efficiency was found to be 91% [60].

El Ouardi et al. used clay as a low-cost, efficient, available, and potential sorbent for nitrate removal from wastewater solutions. The pH, the mass of the adsorbent, the contact time, and the initial solution concentration were found to be relevant parameters for the adsorption processes. The equilibrium of the adsorption process was reached within 3 h, and the removal efficiency diminished with increasing pH value [61]. Red mud is also an efficient adsorption medium for nitrate removal. The nitrate adsorption ability of pristine and activated red mud, respectively, was determined to be 5.858 and 1.859 mmol nitrate/g dry wt. of red mud. The elimination of nitrate with activated red mud, on the other hand, was found to be three times greater than the original form. The adsorption capacity of nitrate with activated red mud was found to be greater than that of the original form. However, it decreases beyond pH 7, and the adsorption steadiness of nitrate ions is achieved in 60 min under these conditions [62].

Amongst several readily available and low-cost materials, rice chaff has respectable features for the adsorption process of nitrate and other wastes. It has abundant floristic fiber, protein, and some efficient groups, e.g., amidogen, hydroxyl, and carboxyl, which can create promising and efficient adsorption routes. About 53% (maximum adsorption) of the entire nitrate was removed within 5 min after the initial start of the test, when the temperature was 400 °C, pH was 3.0, and the amount of chaff was 3 g per liter [63]. Compared with other adsorbents, and from the Q_m_ value (where Q_m_ is the monolayer capacity of the sorbent) of the MCS, one can find that the MCS is an efficient adsorbent material in the nitrate removal from aqueous solutions. The adsorption capacity of NO_3_^−^ onto MCS was affected by many factors such as dosage, agitation time, pH, and temperature. The adsorption was found to be a quick process under these conditions, and the equilibrium was reached at 30 min. The greatest nitrate removal occurs within a pH range of 6.0–12.0 and with 0.2 g of adsorbent. The Q_m_ values of the modified cassava straw residues (MCS) for NO_3_^−^ adsorption decline with increasing temperature. The equilibrium and the kinetics data of the adsorption of nitrate onto MCS were designated by Langmuir and Freundlich isotherm models, respectively, and the kinetics of the adsorption of the nitrate at diverse initial concentrations (20, 50, and 75 mg/L) all agree with a pseudo-second-order equation. The adsorption rates were organized by inter-particle diffusion and each membrane diffusion [64]. Granular Ferric Hydroxide (GFH) is considered a cost-effective, functional, and simply conducted pathway for the nitrate removal from aqueous solutions; it was found that the nitrate in the liquid solution is efficiently uptaken by the GFH adsorbent. The nitrate adsorption rate exhibited an initial upsurge, reaching a plateau at a relatively sluggish rate. Nitrate adsorption was augmented with diminishing initial nitrate concentration and augmented with the adsorbent dosage. The optimum nitrate adsorption in the aqueous solution was detected at a contact time of 90 min and pH = 4.8, and the maximum nitrate removal from wastewater was more than 60% [65].

Modified sugarcane bagasse biochar can serve as an efficient adsorbent material for nitrate removal from aqueous electrolytes. The efficacy of nitrate adsorption reached the best value below basic pH values as compared to the value at a basic pH, owing to the electrostatic interaction between the modified biochar surface positively charged and the nitrate ions. The data elucidate that the equilibrium of adsorption was reached within 60 min, and the empirical results agreed well the Langmuir isotherm model and with a pseudo-second-order kinetic reaction. The maximum adsorption efficiency of these materials for nitrate ions was found to be 28.21 mg g^−1^ [66].

For the removal of nitrate from wastewater, a new adsorbent made of chemically modified hazelnut shells was developed. The adsorption studies were carried out to determine what impact adsorbent concentration, contact duration, starting nitrate concentration, and solution pH had on adsorption. The nitrate removal effectiveness improved as the absorbent quantity increased but declined as the starting nitrate concentration increased. Over a pH range of 2–10, chemically modified hazelnut shells were shown to be successful in nitrate removal, with the greatest quantity of nitrate adsorbed being 25.79 mg g^−1^. It was discovered that a pseudo-second-order model may well represent the kinetics, and the nitrate adsorption process can be regulated by chemisorption. Adsorption/desorption trials in a column validated the absorbent material’s suitability for application many (three) times [67].

## 4. Biological Denitrification

Along with its low cost and great effectiveness, the biological denitrification process is one of the most prevalent nitrogen removal methods [68]. Optional anaerobes, which are required for various organic and inorganic food and energy sources, frequently accompany the biological nitrate removal process. The denitrifiers may be divided into two groups based on this information: autotrophs and heterotrophs. Heterotrophs are microorganisms that need organic substrates to thrive and flourish. Carbohydrates and other organic materials provide them with energy as well. Autotrophs, on the other hand, are microorganisms that use inorganic substances as a source of energy and CO_2_ as a carbon basis [69]. The heterotrophic denitrification (HDN) process has been widely studied for treating wastewater. Nevertheless, exterior carbon foundations might be mandatory for this process due to the low COD/NO^3−^-N ratio (C/N ratio) in the water surface [70], whereas the autotrophic denitrification (ADN) process has been used recently because no exterior based-carbon materials are vital and resulted in less mud compared to the HDN process [71]. In the case of HDN, each liquid and solid formula of carbon-based sources is used, although the aqueous type is more effective for water and wastewater treatment. Among liquid-based-carbon samples, the most well-known ones are ethanol and methanol [72]. On the other hand, ADN uses inorganic carbon-based substrates (bicarbonate or CO_2_) as a food source and depends on electron donors such as hydrogen or reduced sulfur materials for the consumption of energy [73].

Some features of ADN over HDN are the efflux of the destroying influence of some carbon-based compounds, small biomass build-up, and lower sludge fabrication which, results in an easier post-treatment and a reduction in reactor clogging [74]. High-rate ADN using thiosulfate (S_2_O_3_^2−^) was preserved under psychrophilic circumstances in lab-scale FBR with Thiobacillus biofilm. The influence of temperature on the denitrification efficiency of the FBR was noticed by diminishing the temperature from 293 to 276 K. The FBR biofilm efficiency to preserve thiosulfate energetic denitrification at 276 K was checked at diverse hydraulic retention times (HRTs) (5.4, 3, and 1.0 h) and influent NO_3_**^−^** doses (200.0, 600.0 and 1078 mg/L), resulting in an upsurge of the loading rate of nitrogen (NLR) from 0.21 to 3.3 kg N-NO_3_**^−^**/m^3^d. PCR-DGGE exploration demonstrates the control of the sulfur-oxidizing chemolithotrophs T. thioparus and T. denitrificans processes at all the temperatures examined. The FBR process at a temperature as low as 276 K corroborated the bed extension and augmented the dissolved organic carbon (DOC) concentration in the waste but had no important effects on the proficiency of the denitrification process. The study’s originality is highly substantial for managing cold nitrogen-dirtied waters that are poor in organics and confirms that FBR is an effective and potent bioreactor classifier for ADN [75].

The technology of combining micro-electrolysis and biological denitrification (MEBD) employing Fe scrapings and a micro-electrolysis transporter based on activated carbon was enhanced. Under microaerobic conditions, effective instantaneous nitrification and autotrophic denitrification were accomplished, yielding total nitrogen (TN) removal efficiency of 95.3% and a TN elimination load of 0.373 ±  0.11 kgN/ m^3^d. Principal microorganisms were discovered to belong to the Proteobacteria classes β-, α-, and γ−, as well as Nitrospira. Hydrogenophaga and Sphaerotilus were the most common genera in the MEBD reactor. Eighty percent of the TN was removed by autotrophic denitrification. The results validated the functional groupings and the MEBD process’s good functioning [76].

The giant reed (*A. donax*) and licorice (*G. glabra*) demonstrated positive results in applications of drinking water purification as far as the sole physical and chemical supporting material for the denitrifying micro-organism. The outcomes showed that whole nitrate removal was achieved with G. glabra. The efficacy of removal for A. donax was diverse, between 87 and 100%, depending upon the type of process utilized. The small quantity of the used substrates indicates the rare accessibility of carbon-based content in the scanned natural organic materials. The maximum rate of denitrification was gained in the incessant flow systems (4.23 mg N-NO_3_^−^/L h for A. donax and 6.96 mg N-NO_3_^−^/L h for G. glabra). Nevertheless, G. glabra appears to be a superior carbon basis, whereas A. donax seems inexpensive. Owing to their wide accessibility and low prices, these substrates can be utilized as an effective carbon source for the biological route of denitrification [77].

A new halophilic *Vibrio sp*. Y1–5 demonstrated an outstanding nitrogen removal efficiency with no release of the greenhouse gas N_2_ or N_x_O. The optimum circumstances for the reduction of nitrate ions were 1.0%−5.0% salinity, 25.0–35.0 °C temperature, C/N ratio 15–17, and pH 6–9, and the strain displayed effective ammonium removal at extensive ammonium loads (up to 1600 mg/L). Furthermore, Vibrio sp. Y1–5 showed effective prevention of nitrogen loss and could be securely used in aquatic environments [78]. Pseudomonas stutzeri immobilized on microbial cellulose (MC) can be utilized effectually for nitrate elimination. The MC from Acetobacter xylinum was applied as a supporting substance for bacterium immobilization. Owing to its purity and porosity, MC might be reflected as a suitable support for the immobilized adsorbed cells. It was established that the reactor worked well at a neutral pH for the nitrate removal process. Even though a low initial concentration of nitrate ions was preferred for nitrate elimination, the rate of denitrification was increased by increasing the nitrate loading, and the rate of denitrification reached the extreme value of 1.6 kg NO_3_-N m^−3^ day^−1^. This technology has the potential to be used to remove nitrates in contaminated areas due to its high rate of denitrification and outstanding effectiveness in a wide range of circumstances [79].

The sluggish sand filtration technique could successfully remove for the removal of nitrate from drinking water. It is known that most NO_3_-N can be removed from the top level of the sand filter. It was also found that NO_3_-N doses were decreased from an initial concentration of about 22.6 mg/L to lower than the drinking water boundary value at whole filtration rates. It was observed that no noteworthy NO_2_-N growth occurred in the denitrified water at the att filtration rates, except for the uppermost value. It was found that the increase in the rate of filtration from 0.015 to 0.06 m/h had no opposing influence on the effluent of the filter. Nevertheless, 1.0 mg NO_2_-N/l continued in the effluent water at the utmost filtration rate. The sluggish sand filter was incapable of delivering a removal rate of NO_3_-N more than 27.1 g N/m^2^ day [80].

Heterotrophic bacteria separated from the Shiraz municipal wastewater treatment plant were used for the nitrite and nitrate removal from the underground water at a low scale utilizing grape extract as a source of carbon and filamentous media at a temperature of 20 ± 1 °C and a constant pH (7 ± 1). It was found that at retention times of 1, 1.5, 2, and 2.5 h, the rates of nitrate removal were 49.0%, 55.0%, 67.0%, and 67.0%, respectively. Thus, the best retention time was 2 h with a 67% removal rate. The resulting nitrite concentration was approximately 0.001 mg/L NO_3_^−^ at all retention times, which was lower than the standard limit. For that purpose, the use of grape extract as the proper growth of Pseudomonas bacteria and the carbon source in filamentous media significantly increased the removal rate of nitrate [81].

Microbial granules displaying effective nitrate denitrification were developed in a patterning batch reactor by feeding simulated nitrate wastewater with an initial pH of 7.5. At a steady state, 3000 mg L^−1^ NO_3_^−^ (6000 mg L^−1^ NO_3_^−^ in the feed) was quickly and totally denitrified within the first 3 h of the cycle period. Eventually, the biomass granular reactor was appropriately used for denitrifying nitrate wastewater with a pH of 4.0 or 5.0. In situ neutralization in the reactor by the denitrification produced alkalinity along with adaptation and enhancement of microorganisms, permitting denitrification of acidic waters. SEM analysis exhibited the existence of extended rod- and small-rod- or cocci-designed microorganisms on the granules surface produced at pH 7.5 and 4.0. From these data, it can be concluded that the alkalinity produced because of denitrification can be used for the elimination of nitrate-contaminated acidic waters [82].

## 5. Zero Valent Iron

ZVI is well recognized as one of the most reactive and probable compounds utilized for nitrate removal via chemical reduction methods. Nano-scale zero-valent iron (n-Fe^0^) has an active performance for nitrate removal due to its particle size and surface area, but it is not a stable form of iron. It can be slowly oxidized with air and/or water to its hydroxides forms, converting to its larger oxidation states (Fe^2+^, Fe^3+^) [83]. Therefore, ZVI particles should have a distinctive core–shell configuration. The core consists of zero-valent or metallic Fe, and the shell is created with a mixed valent (i.e., Fe^2+^ and Fe^3+^) oxide due to metallic Fe oxidation [84].

### 5.1. Methods for the Synthesis and Characterization of ZVI NPs

In recent decades, numerous routes have been developed to synthesize Fe nanoparticles, modifying the surface features of nanoparticles, and promoting their activity for field delivery and reactions [85]. The most commonly used routine for ZVI synthesis for ecofriendly determinations is the reduction of iron(II) or iron(III) ions in an aqueous solution using sodium borohydride [86].

The core involves ZVI and delivers the reducing power for the reactions. The shell is mainly iron hydroxides/oxides designed from the oxidation of ZVI. The shell delivers positions for chemical complex development (e.g., chemical adsorption). Ibrahim et al. have suggested that the ZVI nanoparticles were prepared in a flask reactor in an ethanolic solution with three open necks containing Fe(III) and sodium borohydride based on the following equation [87]:

6NaBH_4_ + 2FeCl_3_ + 18H_2_O → 2Fe^0^ + 6B(OH)_3_ + 21H_2_ + 6NaCl (2)

In this method, a specified weight of iron (III) chloride (FeCl_3_.6H_2_O) was dissolved in a mixture of 1/4 (*v*/*v*) water/ethanol and stirred well. Then, 0.1 mol/L sodium borohydride solution was prepared. For the good growth of Fe nanoparticles, excess sodium borohydride is required. The sodium borohydride solution is poured into a burette and added slowly drop by drop into FeCl_3_.6H_2_O solution with energetic stirring. After the first drop of borohydride solution leads to the appearance of black solid particles, the residual borohydride solution is added totally to increase the reaction rate. The mixture is stirred further for several minutes after adding the complete borohydride solution. The vacuum filtration technique is utilized to isolate the solid black Fe metal nanoparticles from the liquid phase. The resulting solid particles are thoroughly washed many times with small quantities of absolute ethanol to eliminate the adsorbed H_2_O. It should be taken into account that this washing route is possibly the key stage of preparation since it inhibits the quick oxidation of ZVI NPs. The prepared ZVI NPs are finally dried in an oven at 50 °C for 24 hrs. For storing, a small amount of ethanol is added to protect the nZVI from oxidation [87]. In other work, nanoscale zero-valent iron (nano ZVI) was prepared by adding a 1:1 volume ratio of FeCl_3_.6H_2_O and NaBH_4_, and then the solution was intensively mixed under 25 °C for 5 min according to this equation:

4Fe^3+^ + 3BH_4_^−^ + 9 H_2_O → 4Fe^0^ + 3H_2_BO^3−^ + 12 H^+^ + 6H_2_ (3)

Then nZVI is filtered using Whatman filter paper and washed several times with deionized H_2_O to develop rid of extreme borohydride [88,89].

Another researcher prepared ZVI nanoparticles by dissolving 33.77 g of FeCl_3_·6H_2_O in 250 mL of deionized water (0.5 M of Fe^(III)^). The resultant solution was moved to a 500 mL glass beaker. Then, 9.4 g of sodium borohydride powder was dissolved in 250 mL deionized H_2_O and added gradually to the iron (III) chloride solution utilizing a peristaltic pump with a flow rate of about 10.0 mL per min. The mixture was stirred mechanically at 250 rpm throughout the addition of NaBH_4_. The mixing was stationary for 15 min after the complete NaBH_4_ solution was added. No surface active agent was added to keep the dispersion of Fe particles. The mixture was centrifuged at 4000 rpm for 30 min, and the produced solid was washed twice with bidistilled H_2_O. The synthesized wet NZVI was applied directly for batch experimentations [90]. ZVI NPs can be correspondingly prepared by using ferrous sulfate instead of ferric chloride as follows.

Twenty grams of Fe_2_SO_4_.7H_2_O was dissolved in a 2:3 (*v*/*v*) ethanol/deionized water mixture and fully dissolved by stirring at 650 rpm for 10 min. Before the experiment, deionized (DI) H_2_O was removed with refined nitrogen gas for 15 min to eliminate the dissolved oxygen. After that, 2 g of NaBH_4_ was added to 50 mL of bidistilled H_2_O. The NaBH_4_ solution was added dropwise to aqueous Fe salt at 5 mL/min and vigorously stirred at 650 rpm. All procedure was accomplished under an N_2_ atmosphere, whereas the external surface of the reaction vessel was cooled down with ice to prevent the oxidation of ZVI NPs. Throughout this method, the solution sluggishly changed to a black color. The produced black particles were washed by deionized water saturated with N_2_ and then three times at least by absolute ethanol. Lastly, the prepared ZVI NPs were desiccated in a desiccator. The dried particles were utilized for further characterization [91,92,93].

**Figure 1:**
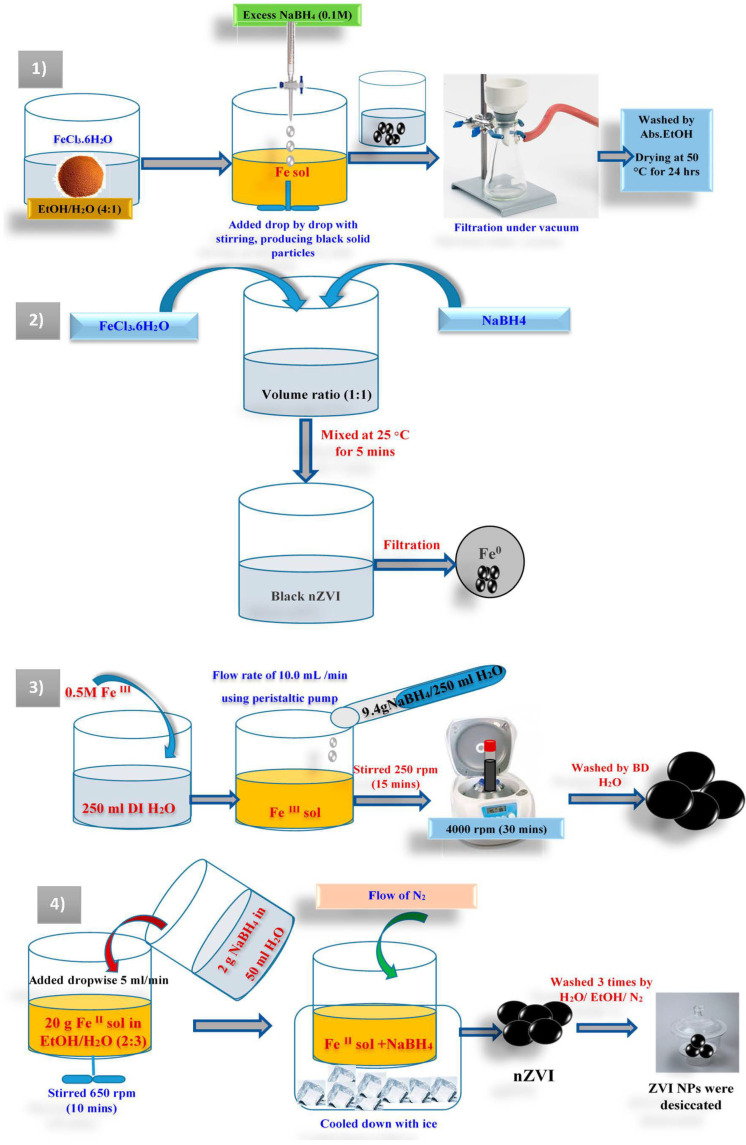
A representative scheme introduces a summary of the mentioned synthetic procedures.

Another approach exposed a typical route, in which a stoichiometric molar ratio of NaBH_4_ to ferric chloride (FeCl_3_·6H_2_O) (1:2), a definite volume of deionized water and absolute ethanol (1:1) was gradually blended. The resulting blend was stirred at 350 rpm for 30 min. Then, the mixture was settled for further 30 min. The obtained solid sample was collected using a centrifuge followed by washing with deionized (DI) water and ethanol four times. Then, the sample was dried under a vacuum for further characterizations [94]. A summary of the mentioned synthetic procedures is introduced in a representative scheme (Figure 1).

### 5.2. Characterization of ZVI Nanoparticles

ZVI nanoparticles can be categorized by many tools, including TEM, SEM, XRD, MS, and XPS. These techniques can provide strong evidence for the presence of nZVI [95].

#### 5.2.1. XRD

This technique gives knowledge about the crystalline construction of the material. By using this information from the crystallographic planes of known materials, it is possible to characterize the chemical composition of unknown substances [89,96]. XRD examination established that nano ZVI was synthesized from the borohydride reduction method. Furthermore, XRD also showed the presence of crystalline building of the prepared particles (Figure S1; Supporting information) [89].

#### 5.2.2. TEM

**Figure 2:**
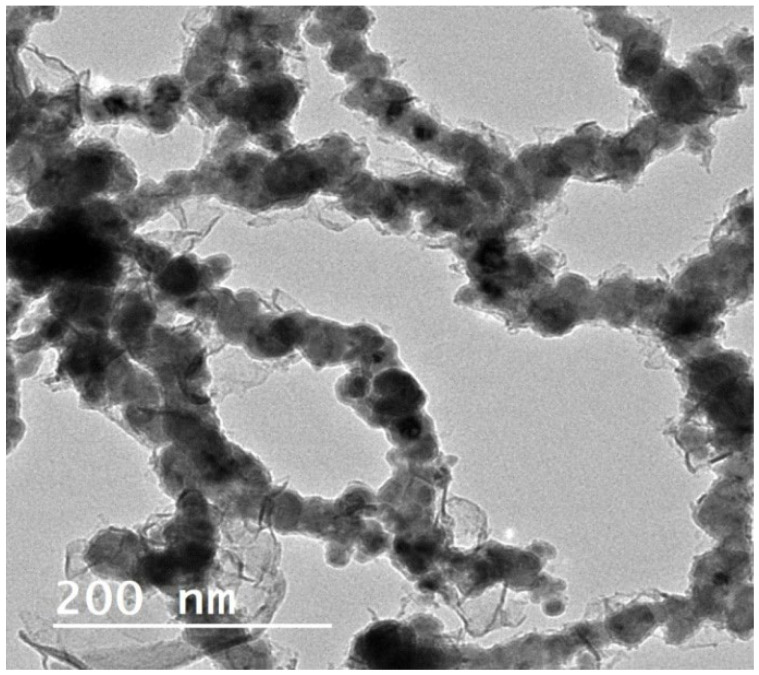
TEM image of ZVI nanoparticles.

TEM allows the identification of the smallest particles whose size is between 5 nm and 20 nm [97]. Using this technique, samples were prepared by the deposition of some drops of a diluted ethanol solution of ZVI NPs onto a substrate of a carbon layer. It was found that the average size was 26.40 nm with a standard deviation (SD) of 16.90 nm (Figure 2) [98,99].

#### 5.2.3. Size and Size Distribution

An acoustic spectrometer can also determine the size distribution and particle size. This spectrometer uses the sound pulses transmitted through a suspension particle to detect the characteristics of suspended particles [100]. The size range was found to be in the range from 5 nm to 1000 μm, and for high accuracy, a concentration particle of at least >1 wt% is recommended [101].

#### 5.2.4. SEM

**Figure 3:**
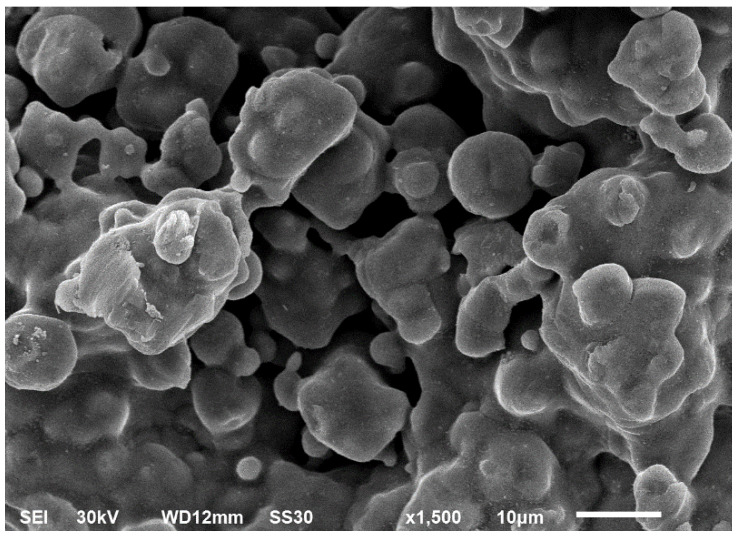
SEM micrograph of ZVI nanoparticles.

SEM is used to measure the texture and morphology of crystal development and can also be ultimately utilized to determine the particle size. Results indicate that the prepared nZVI particles show a dendritic structure. Most of the nZVI particles are in the nanoscale [91,96,101]. The micrograph resulting from SEM examination demonstrates the spherical and uniform morphology of the green ZVI NPs. Green ZVI NPs were established to be very close to each other due to their strong magnetic force and their high concentration (Figure 3) [94].

#### 5.2.5. UV–Vis Spectroscopy

The UV–Vis spectrum of the suspended ZVI NPs in 0.8% CMC exhibited absorption maxima at 235.0 nm [98]. This maximum absorption is characteristic of ZVI.

#### 5.2.6. Zeta-ζ Potential

The ζ-Potential of the ZVI NPs was measured by an acoustic spectrometer. The charged particles’ fluctuation in an acoustic field provides an alternating electric current and electrical fields, which can then be utilized to compute the potential of the particle surface [102]. The charge of iron nanoparticles’ surface is frequently categorized by the ζ-potential, which is distinguished as the electric potential at the shear interface compared to that in the distant bulk solution. ζ-potential or surface charge is the main feature determining the flexibility of particles in an electrical field (Figure S2; Supporting information) [99].

## 6. Removing Nitrate Ions Using Zero-Valent Iron Nanoparticles

The chemical reduction process of nitrate by using nZVI, which is characterized by high surface reactivity, large surface areas (particle size < 100 nm), low-cost process, and non-toxicity, is an emerging environmental technology [103]. This process is considered a strongly exergonic reaction and has long been known to occur [104].

It is well-known that in aqueous solutions, ZVI (Fe^0^) is freely oxidized to ferrous ion (Fe^+2^) by several reagents. Under anaerobic systems, H^+^ is a simple electron acceptor that will be included in the reaction. Consequently, the corrosion process of ZVI in the anaerobic system of Fe^0^–H_2_O can be designated by the following equation [105]:

Fe^0^ + 2H_2_O → Fe^+2^ + H_2_ + 2OH^−^ (4)

However, under aerobic systems, the dissolved oxygen (DO) can play an important part as the electron acceptor in the half-reaction of the cathodic process. In that situation, the prime reaction produces only OH^−^ and not H_2_ [106].

2Fe^0^ + O_2_ + 2H_2_O → 2Fe^+2^ + 2OH^−^ (5)

The mechanism of the reaction between nZVI and nitrate is classified as a factual redox reaction. Numerous studies showed that the final yields of the nitrate chemical reduction by nZVI could be NH_3_ or N_2_, depending on the reaction conditions. However, the principal product of this reaction is ammonium ion [107].

4Fe^0^ + NO_3_^−^ + 7 H_2_O → 4Fe^+2^ + NH_4_^+^ + 10 OH^−^ (6)

In the reduction route by nZVI, the pH of the medium is an essential factor affecting the reaction kinetics, showing that Fe hydroxides participate in stabilizing the pH by consuming OH^−^ ions. In addition, the presence of hydroxide ions affects the free surface coverage by controlling the redox-reaction rate [108].

For batch experiments, 500 mL of nitrate solution at concentrations of 30 and50 mg/L NO_3_-N and initial pH of 4, 7, and 10 was kept in a glass beaker, and freshly synthesized ZVI NPs of concentrations (1000, 500, or 200 mg/L) were mixed. The solution was then stirred by a jar experiment device at a 300 rpm mixing rate. The glass beakers were removed from the jar experiment at break times of 10.0, 20.0, 30.0, and 60.0 min. On some occasions, 20 mL of the aqueous medium was filtered via a membrane filter (0.45 m) to isolate ZVI NPs. The residual nitrate dose (NO_3_**^−^**) in an aqueous medium was measured with a spectrophotometer [109].

### 6.1. Effect of Nitrate Ions Dose on the Reduction Process by nZVI

It was found that the reduction rates of nitrate for 30 mg/L and 50 mg/L NO_3_^−^ increased to 66.50% and 65.70%, respectively, after 10 min. However, after this time, the reduction rate improved marginally such that after 60 min, the rate of reduction reached 78.3% (6.5 mg/L NO_3_) and 79.98% (10.01 mg/L NO_3_^−^), respectively, for 30 mg/L and 50 mg/L NO_3_^−^ [109].

Ruangchainikom et al. used CO_2_ gas with Fe^0^ for nitrate removal. The initial concentrations of the nitrate were 6.6, 12, 22.6, 33.4, and 46.2 mg/L, which declined to 0.70 (90.0% removed), 1.1 (90% removed), 3.0 (87% removed), 9.4 (71% removed), and 13.6 mg/L (70% removed), respectively. Beyond this, the nitrate concentration was still fixed until a definite acute point was reached. After this acute point, the residual nitrate concentration continued to upsurge. This may be attributed to insufficient Fe^0^ in the reaction system [110].

### 6.2. Influence of the pH on NO_3_^−^ Removal

Comprehensive investigations have shown that the pH is a significant restriction affecting the NO_3_ reduction employing ZVI. Various studies showed that the rate of nitrate elimination was inversely associated with the pH of the solution. It was stated that the ZVI interface rests at lower pH values. Accordingly, NO_3_ ions can be removed totally and quickly, whereas when the pH is > 6.5, a small quantity of nitrate reduction is detected in unbuffered systems compared to substantial removal in buffered systems. Additionally, it was noticed that strong acidic mediums are more favorable for nitrate reduction using ZVI [111]. Moreover, when normal Zeolite-reinforced ZVI nanoparticles were used for nitrate removal, the removal process was extremely reliant on the pH of the medium. The increase in H^+^ ions concentration facilitates the electron transfer among nitrate ions and nZVI, and can accordingly inhibit the formation of iron(II) and iron(III) precipitate on the nZVI surface [112]. Shima Ziajahromi et al. suggested that the preliminary pH level has a harmful influence on reducing nitrate ions. It was found that when the pH increased from 4.0 to 10.0, the reduction potentials became lesser. When the pH was 4, around 80.0% of nitrate ions were reduced in 1.0 h, while the reduction potentials declined to 64 and 70% for pH levels of 10 and 7, respectively. This proposes that acidic conditions could be promising in nitrate adsorption using ZVI NPs [109].

### 6.3. Effect of nZVI Dosages

Daniela V. Lopes et al. studied the effect of ZVI dosage by using concentrations of 2000, 4000, 10,000, and 20,000 mg/L of ZVI for the NO_3_ reduction. In an acid solution at pH 2, using higher dosages of ZVI led to decreased nitrate concentrations at the end of the reduction process. The complete reduction of nitrate (99.9% of nitrate reduction) could be attained with 10,000 mg/L after 3 h and 20,000 mg/L after 4.5 h, whereas, with 2.0 g/L dosages, less ZVI was available for the chemical reduction, resulting in 34.0 mg/L of nitrate (78.7% of nitrate reduction) [113].

Jia-Chin Hsu et al. found that when applying a dosage of about 0.5 g of nZVI, the nitrate removal efficiency was 23% in the absence of CO bubbling. However, when 1.0 g was added, the nitrate-reducing effect appeared to be negligible. To put it another way, the higher dose of nZVI, the lower the nitrate removal. This might be because of aggregation in the nZVI preparation, which results in a larger particle size. Another might be the carbonic acid factor generated from CO bubbling [114]. Scientists have recommended other adsorbent materials to remove harmful nitrate anions from potable water. The optimum conditions for different adsorbents to remove harmful nitrate anions from potable water are recorded in Table 1 [115,116,117,118,119,120,121,122,123,124,125,126,127,128].

## 7. Conclusions

The existence of nitrate ions in drinking water signifies a substantial hazard to humans. This hazard could be characterized by decreasing nitrate to nitrite, which participates in the oxidation process of common hemoglobin to methemoglobin, causing methemoglobinemia. Many researchers have presented a variety of approaches for eliminating nitrate from potable water. Sulfamic acid with Zn metal is utilized for the removal process. Electrochemical techniques are also used for this purpose, which is dependent on several parameters such as current density, electrolyte, temperature, and pH of the electrolyte solution. Many metals and alloys, such as aluminum, iron, copper, copper-zinc, and stainless steel, have been used as cathode materials. Aluminum, titanium, and iron cathodes have high efficiency in removing nitrate, which may be due to their greater electron-donating capacity. Another technique is the adsorption process, which is a physical phenomenon. In this process, the substances with high surface capacity are used as adsorbents for the nitrate. Activated carbon is considered one of the most used adsorbents and is the most significant material to remove environmental pollution. The biological technique is one of the most promising methods for various removal processes due to its high efficiency and low cost. In this process, the researchers used a variety of microbial agents, leading to the high efficiency of nitrate removal. Autotrophic denitrification contributes to about 80% of nitrate removal. Among all previous compounds used for nitrate removal, ZVI is the most probable and sensitive compound utilized for removing nitrate via chemical reduction methods. ZVI can be prepared by reducing ferric or ferrous compounds using a reducing agent such as borohydride, and the resulting ZVI can be characterized by many tools, including SEM, TEM, and XRD. These techniques can be used to provide strong evidence for the presence of nZVI. Researchers have removed 99.9% of nitrate using ZVI, depending on reaction parameters such as ZVI dose, pH, and the nitrate content in the solution.

## Figures and Tables

**Table 1:** Optimum parameters for nitrate removal by different adsorbents.

No.	Adsorbents	Adsorbed Amount (mg/L) for 1 g of Absorbent	Nitrate Dose(mg/L)	Contact Time/min	pH	*T*/°C	Refs.
**1**	Activated Carbon Derived from Rice Straw	10	50	1440	3–9	25	[49]
**2**	Modified granular activated carbon	5	40	120	7	30	[51]
**3**	Activated carbon and composite of Fe_2_O_3_ nanoparticles and activated carbon	23–107	66–234	120	3–8	30	[52]
**4**	Zeolite-Supported Zero-Valent Iron Nanoparticles	10	100	1440	5.5	25	[112]
**5**	Sulphuric acid Treated carbon cloth	12.4	115	60	7	25	[115]
**6**	Powdered activated carbon	62	-	60	5	25	[116]
**7**	Carbon nanotubes	155	-	60	5	25	[116]
**8**	Untreated coconut granular activated carbon	0.17	1.0	120	5.5	25	[117]
**9**	Zinc chloride treated coconut granular activated carbon	10.2	5–200	120	5.5	25	[117]
**10**	Coconut shell activated carbon	16.5	5–200	-	2–4	30	[118]
**11**	Bamboo-charcoal	6.44	-	-	2–4	30	[118]
**12**	Bamboo powder charcoal	1.25	-	120	5.4	10	[119]
**13**	Halloysite	0.54	0–10	1020	5.4	25	[120]
**14**	HDTMA modified QLD-bentonite	12.8	100	1020		25	[120]
**15**	Calcined hydrotalcite-type compounds	61.7	12.7–236	1440	8.5	25	[121]
**16**	Layered double hydroxides	20	0–1000	240	5	21	[122]
**17**	Chitosan coated zeolite	37.2	10–3100	4320	5	20	[123]
**18**	Chitosan hydrobeads	92.1	1–1000	1440	5	30	[124]
**19**	Chitosan beads	90.7	25–1000	1440	-	30	[124]
**20**	Conditioned cross-linked chitosanbeads	104.0	25–1000	1440	-	30	[124]
**21**	Pure alkaline lignin	11.16	1–30	2880	-	30	[125]
**22**	Sugarcane bagasse	8.74	1–30	2880	-	30	[126]
**23**	Pure cellulose	8.31	1–30	2880	-	30	[127]
**24**	Rice hull	8.18	1–30	2880	-	30	[128]

## Data Availability

The raw/processed data generated in this work are available upon request from the corresponding author.

## Supplementary Materials

The following supporting information can be downloaded at: https://www.mdpi.com/article/10.3390/molecules27082552/s1, Figure S1: XRD analysis of ZVI nanoparticles; Figure S2: Zeta-ζ potential measurements of ZVI nanoparticles.

## Abbreviations

SEM	Scanning electron microscope
ZVI NPs	Zero-valent iron nanoparticles
TEM	transmission electron microscope
MS	Mössbauer spectroscopy
XRD	X-ray diffraction
FT-IR	The Fourier transform infrared spectroscopy
CMC	Carboxymethyl cellulose
XPS	X-ray photoelectron spectroscopy
EDX	Energy-Dispersive X-Ray
LPR	Linear-polarization resistance
CV	Cyclic voltammetry
FBR	Fluidized-bed reactor
CA	Chronoamperometry
SMZ	Surfactant-modified zeolites
COD	Chemical oxygen demand
GFH	Granular Ferric Hydroxide
MC	Microbial cellulose
MCS	Modified cassava straw
DOC	Dissolved organic carbon
CPB	Cetyl pyridinium bromide
