# Supplementary material for: Removal of the Harmful Nitrate Anions from Potable Water Using Different Methods and Materials, including Zero-Valent Iron
Source: Molecules. 2022 Apr 14;27(8):2552. doi: 10.3390/molecules27082552 (PMC9031846; doi:10.3390/molecules27082552)
Supplement: Supplementary file 1 [file molecules-27-02552-s001.zip › molecules-1637072-Supplementary Materials.pdf]

Removal of the Harmful Nitrate Anions from Potable Water Using Different Methods and Materials, including Zero-Valent Iron

Hany M. Abd El-Lateef [1,2,*], Mai M. Khalaf [1,2], Alaa El-dien Al-Fengary [2] and Mahmoud Elrouby [2,3,*]

[1] Department of Chemistry, College of Science, King Faisal University, P.O. Box 400, Al-Ahsa 31982, Saudi Arabia; mmkali@kfu.edu.sa
[2] Chemistry Department, Faculty of Science, Sohag University, 82425 Sohag, Egypt; alaa.eldaly2@yahoo.com
[3] Faculty of Science, King Salman International University, 46612 Sinai, Egypt
* Correspondence: hmahmed@kfu.edu.sa or Hany_shubra@science.sohag.edu.eg (H.M.A.E.-L.); dr_mahmoudelrouby@science.sohag.edu.eg or Mahmoud.elrouby@ksiu.edu.eg (M.E.)

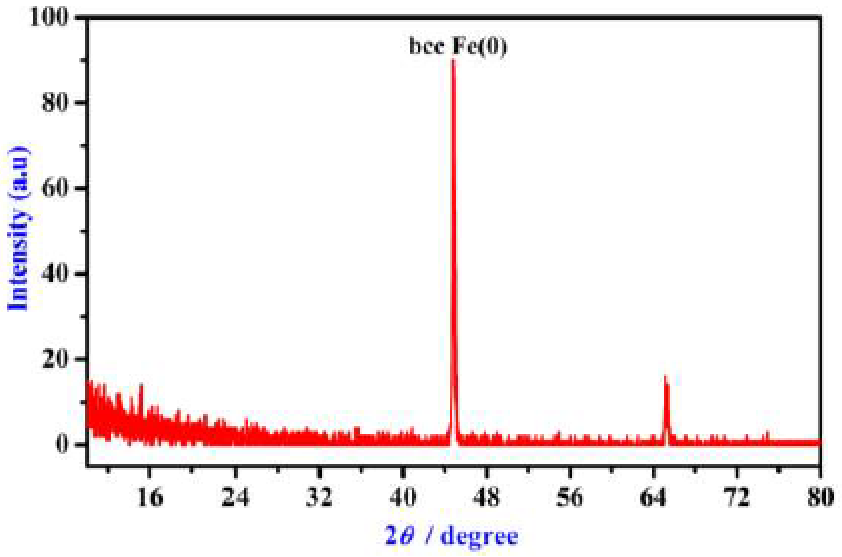

Figure S1. XRD analysis of ZVI nanoparticles.

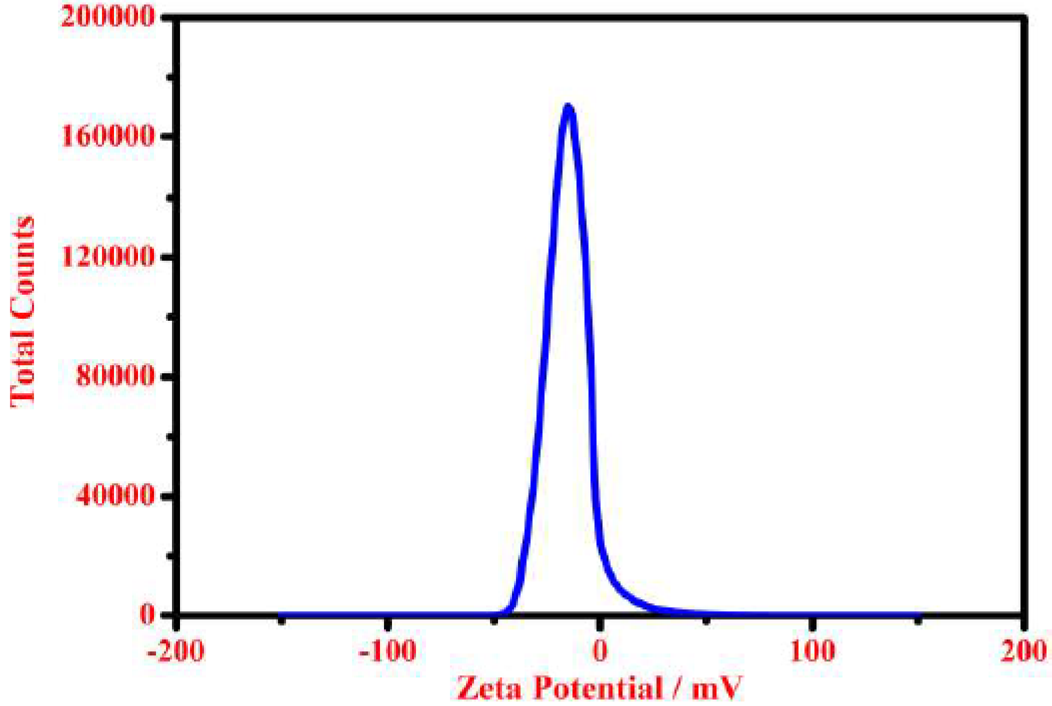

Figure S2: Zeta-ζ potential measurements of ZVI nanoparticles.
